# Antibacterial effect of essential oils and their components against *Xanthomonas arboricola* pv. *pruni* revealed by microdilution and direct bioautographic assays

**DOI:** 10.3389/fcimb.2023.1204027

**Published:** 2023-06-14

**Authors:** Judit Kolozsváriné Nagy, Ágnes M. Móricz, Andrea Böszörményi, Ágnes Ambrus, Ildikó Schwarczinger

**Affiliations:** ^1^ Plant Protection Institute, Centre for Agricultural Research, Eötvös Lóránd Research Network, Budapest, Hungary; ^2^ Department of Pharmacognosy, Faculty of Pharmaceutical Sciences, Semmelweis University, Budapest, Hungary; ^3^ Plant Health Bacteriological Diagnostic National Reference Laboratory, Food Chain Safety Laboratory Directorate, National Food Chain Safety Office, Pécs, Hungary

**Keywords:** *Xanthomonas arboricola* pv. *pruni*, essential oil, high-performance thin-layer chromatography, SPME-GC/MS, direct bioautography, broth microdilution assay, antibacterial efficacy

## Abstract

Bacterial spot of stone fruits caused by *Xanthomonas arboricola* pv. *pruni* (*Xap*) is one of the most significant diseases of several *Prunus* species. Disease outbreaks can result in severe economic losses while the control options are limited. Antibacterial efficacy of essential oils (EOs) of thyme, cinnamon, clove, rosemary, tea tree, eucalyptus, lemon grass, citronella grass, and lemon balm was assessed against two Hungarian *Xap* isolates. The minimal inhibitory concentration (MIC) was determined by broth microdilution assay and for the identification of active EOs’ components a newly introduced high-performance thin-layer chromatography (HPTLC)-*Xap* (direct bioautography) method combined with solid-phase microextraction-gas chromatography/mass spectrometry (SPME-GC/MS) was applied. All EOs inhibited both bacterium isolates, but cinnamon proved to be the most effective EO with MIC values of 31.25 µg/mL and 62.5 µg/mL, respectively. Compounds in the antibacterial HPTLC zones were identified as thymol in thyme, *trans*-cinnamaldehyde in cinnamon, eugenol in clove, borneol in rosemary, terpinen-4-ol in tea tree, citral (neral and geranial) in lemon grass and lemon balm, and citronellal and nerol in citronella grass. Regarding active compounds, thymol had the highest efficiency with a MIC value of 50 µg/mL. Antibacterial effects of EOs have already been proven for several *Xanthomonas* species, but to our knowledge, the studied EOs, except for lemon grass and eucalyptus, were tested for the first time against *Xap*. Furthermore, in case of *Xap*, this is the first report demonstrating that direct bioautography is a fast and suitable method for screening anti-*Xap* components of complex matrices, like EOs.

## 1 Introduction

The bacterial spot of stone fruits and almond is a significant disease of many *Prunus* species with a large and increasing distribution area. Its causative agent, *Xanthomonas arboricola* pv. *pruni* (Smith, 1903; Vauterin et al., 1995) (abbreviated as “*Xap*” hereafter) is included in the A2 list of pests by the European and Mediterranean Plant Protection Organization (EPPO, 2023). *Xap* was first identified in Hungary in 2004 (Németh, 2005). Its spread was successfully inhibited due to the applied quarantine measures, but from 2016 the pathogen was re-detected on different hosts and from several Hungarian locations (Schwarczinger et al., 2017; Schwarczinger et al., 2018; Kolozsváriné Nagy et al., 2020). *Xap* poses a real threat to its economically important hosts (peach, nectarine, apricot, Japanese plum, and almond) especially in areas where the climate is humid and warm during the growing season (Battilani et al., 1999). This pathogen induces water-soaked spots on the surface of leaves, fruits, and shoots that can become brown and necrotized (Civerolo and Hatting, 1993). Its significance lies in the fact that severe leaf infections lead to diminished vitality of the trees and the highly infected fruits are simply unmarketable, while twig cankers combined with early defoliation may result in complete destruction of the plant (EPPO, 2006). The management of this disease is mainly limited to prevention – *i.e.* the certification of propagation material, the use of less susceptible cultivars (Lamichhane, 2014) or disease forecasting models (Battilani et al., 1999; Morales et al., 2018) – and the application of copper-based pesticides (Osdaghi, 2022) and oxytetracycline (OTC) in the USA (Vidaver, 2002). However, most peach, apricot, and Japanese plum genotypes are susceptible to the bacterium (Garita-Cambronero et al., 2018) and multiple copper treatments can result in not only phytotoxicity but the development of copper tolerant or resistant plant pathogenic bacterial strains (Giovanardi et al., 2017; Cox et al., 2022). As for the other chemical pesticide, OTC resistance has been reported in *Xap* strains in peach orchards of the southeastern USA (Herbert et al., 2022). Therefore, the study of alternative control measures like the usage of essential oils (EOs) is of high priority.

EOs are natural complex mixtures of botanical origin carrying a distinctive scent, or essences of the originating plants, which have a history of several thousand years (EFEO/IFRA, 2015). Compared to synthetic pesticides, natural ones have beneficial features including biodegradability, target specificity, and posing no or lower risk to the environment or human health (Walia et al., 2017). The therapeutic use of biologically active volatile plant compounds includes clinical (Buckle, 2015), food preservative (Corrêa and Ferreira, 2022), and plant protection (Assadpour et al., 2023) applications. The great potential of EOs as biopesticides is based on their herbicidal (Li et al., 2020; Yasar et al., 2021), insecticidal (Isman et al., 2011; Ebadollahi et al., 2022), acaricidal (Camilo et al., 2017; Nwanade et al., 2021), nematicidal (Avato et al., 2017; Catani et al., 2023), and antimicrobial effects (Božik et al., 2017; Assadpour et al., 2023), and has been well demonstrated by usage of commercialized bioinsecticides (Isman, 2020). A variety of EOs has shown effectiveness against several *Xanthomonas* species (Horváth et al., 2004; Huang and Lakshman, 2010; Bajpai et al., 2011; Lucas et al., 2012; Móricz et al., 2015a; Móricz et al., 2015b; Gormez et al., 2016; Mirzaei-Najafgholi et al., 2017; Singh et al., 2017; Kumar et al., 2021; Hakalová et al., 2022; Montesinos et al., 2023) and especially against *Xap* (Biavati et al., 2004; Mahmoudi et al., 2010; Zhao et al., 2011; Montesinos et al., 2023).

Among *in vitro* antibacterial susceptibility assays, routine well and disc diffusion methods are preferably used for the examination of polar components due to the limited diffusion of lipophilic compounds to the agar (Rios et al., 1988; Móricz and Ott, 2016). Therefore, the commonly used dilution methods and bioautography are more suitable for the determination of antibacterial activity of EOs that are mixture of volatile, complex, and viscous substances generally with low solubility in water. Direct bioautography (DB), the combination of high-performance thin-layer chromatography (HPTLC) with antibacterial assays, is a sensitive, fast (it takes less than 3 h), and reproducible high-throughput screening protocol for assessing the antibacterial effects of EOs, without diffusion or solubility limits (Horváth et al., 2013; Dewanjee et al., 2015; Móricz and Ott, 2016). Furthermore, DB facilitates detection of HPTLC inhibition zones allowing estimation of the antibacterial effects of individual separated compounds that can be characterized and identified by various HPTLC hyphenations *e.g.* spectroscopy and mass spectrometry (Móricz and Ott, 2023).

Due to the lack of registered antibacterial compounds effective against *Xap*, the aim of the present study was (i) the survey of antibacterial impacts of the EOs of thyme, cinnamon, clove, rosemary, tea tree, eucalyptus, lemon grass, citronella grass, and lemon balm on *Xap*; and (ii) the identification of the tested EOs’s active components by the HPTLC-*Xap* method combined with solid-phase microextraction-gas chromatography/mass spectrometry (SPME-GC/MS).

## 2 Methods

### 2.1 Bacterial isolates

The bacterial isolates used: *Xap*3, isolated from *Prunus salicina* Lindl. in 2004 (No. 2369-12/2004, J. Németh, Plant Health Bacteriological Diagnostic National Reference Laboratory, Pécs, Hungary) (Németh, 2005) and *Xap*G2, identified from *P. armeniaca* L. cv. Bergecot in 2016 (No. XapHU1, I. Schwarczinger, Plant Protection Institute, ARC, Budapest, Hungary) (Schwarczinger et al., 2017). Nutrient broth (NB) and nutrient agar (Biolab, Budapest, Hungary) were used for propagation and maintenance. The bacterial cell concentration was synchronized to an optical density (OD) of 0.3 at 600 nm and diluted hundredfold (1 × 10^5^ CFU/mL) in NB.

### 2.2 Materials

Aluminum foil-backed HPTLC silica gel 60 F_254_ layer was purchased from Merck (Darmstadt, Germany). Solvents for HPTLC experiments were of analytical grade from Molar Chemicals (Halásztelek, Hungary). Vanillin was from Reanal (Budapest, Hungary), and concentrated sulfuric acid (96%) from Carlo Erba (Milan, Italy). Chloramphenicol (purity ≥ 98%) was acquired from Sigma-Aldrich (Budapest, Hungary), and vital dye reagent 3-[4,5-dimethylthiazol-2-yl]-2,5-diphenyltetrazolium bromide (MTT) from Carl Roth (Karlsruhe, Germany).

### 2.3 Essential oil samples

EOs of *Thymus vulgaris* L. (thyme), *Cinnamomum ceylanicum* Nees. (cinnamon), *Syzygium aromaticum* (L.) Merr. et Perry (clove), *Rosmarinus officinalis* L. (rosemary), *Melaleuca alternifolia* (Maiden et Betche) Cheel (tea tree), *Eucalyptus globulus* Labill. (eucalyptus), and *Cymbopogon citratus* (DC.) Stapf. (lemon grass) were obtained from Herbaria (Budapest, Hungary), and *Cymbopogon nardus* (L.) Rendle (citronella grass) was from AroMax (Budapest, Hungary). *Melissa officinalis* L. (lemon balm) was purchased from two manufacturers: Primavera Life (Oy-Mittelberg, Germany) and Olivia Natural (Budapest, Hungary). All EOs were extracted by hydrodistillation. The EO constituents thymol, *trans*-cinnamaldehyde, eugenol, citral (neral and geranial), and citronellal were from Sigma-Aldrich, while borneol, and terpinen-4-ol from Molar Chemicals. The EOs and the active compounds were dissolved in absolute ethanol (50 mg/mL and 20 mg/mL, respectively), and stored at - 20°C in the dark.

### 2.4 HPTLC – *Xap* direct bioautography

Samples dissolved in ethanol were applied at an 8-mm distance from the bottom as 5-mm bands and 8–10-mm track distance onto the HPTLC plate using the Automated TLC Sampler (ATS3, CAMAG, Muttenz, Switzerland). Separation was performed with *n*-hexane – ethyl acetate 9:1 *V*/*V* mobile phase up to a migration distance of 70 mm (Twin Trough Chamber 20 cm × 10 cm, CAMAG). The segments of the dried chromatograms were documented with a digital camera (Cybershot DSC-HX60, Sony, Neu-Isenburg, Germany) at visible light after derivatization with vanillin-sulfuric acid reagent (400 mg vanillin, 100 mL ethanol, and 2 mL concentrated sulfuric acid; heated at 110°C for 5 min), or bioassays (DB) using the two *Xap* isolates.

**Figure 1:**
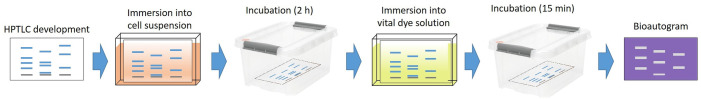
The steps of direct bioautography (HPTLC-*Xap* assay).

The steps of DB are outlined in **Figure 1**. Test bacteria were grown in NB at 28°C on an orbital shaker (130 rpm) to reach a late exponential phase (OD_600 _= 1.2). Developed and dried layers were dipped into bacterial cell suspensions and put into an incubator for 2 h (100% humidity at 28°C), followed by immersion into an aqueous MTT solution (1 mg/mL) to visualize the bioautogram. After an additional 15-min incubation the antibacterial compounds are revealed as bright zones against the darker background (yellow MTT is reduced to bluish MTT-formazan by the dehydrogenases of vital cells).

### 2.5 Broth microdilution assay

Minimal inhibitory concentration (MIC) is defined as the lowest concentration of an antimicrobial agent which prevents the growth of bacteria after 20 h. MIC value of the tested EOs (50 mg/mL in ethanol) and their isolated components (20 mg/mL in ethanol) was determined against the two *Xap* isolates by broth microdilution assay (BMA). Chloramphenicol (1 mg/mL) was used as positive and ethanol as negative control. Ethanolic two-fold dilution series of the samples were prepared in duplicate and 3 µL of each was mixed with 147 µL of bacterial cell suspension (1 × 10^5^ CFU/mL) in 96-well flat-bottom sterile microtiter plates (Wuxi Nest Biotechnology Co., Ltd., Jiangsu, China). In case of the isolated components, an additional concentration of 7.5 mg/mL was also tested. The incubation took place at 28°C for 20 h by shaking at 900 rpm with a PHMP Twin Microplate Shaker-Incubator Thermoshaker (Grant Inc., Beaver Falls, PA, USA). Values of optical density assays at 600 nm were recorded by a Labsystems Multiscan MS 4.0 microplate reader spectrophotometer (Thermo Scientific, Waltham, MA, USA) at zero time point and after the incubation period. Subtraction of the background indicated the rate of cell multiplication. The MIC values were determined as the lowest concentrations of tested samples that completely inhibited bacterial growth. The experiment was repeated twice.

### 2.6 SPME–GC/MS

For identification of the bioactive compounds, HPTLC zones were eluted with ethanol by the use of a TLC-MS Interface (CAMAG), and the eluates were collected into vials (20 mL headspace) sealed with a silicon/PTFE septum and transferred along with the EOs to the SPME-GC/MS analysis. Sample preparation using the static headspace SPME technique was carried out with a CTC Combi PAL (CTC Analytics AG, Zwingen, Switzerland) automatic multipurpose sampler using a 65 μM StableFlex carboxen/polydimethylsiloxane/divinylbenzene (CAR/PDMS/DVB) SPME fibre (Supelco, Bellefonte, PA, USA). After a 5-min incubation at 100°C, extraction was performed by exposing the fibre to the headspace of a 20 mL vial containing the sample for 10 min at 100°C. The fibre was then immediately transferred to the injector port of the GC/MS, and desorbed for 1 min at 250°C. The SPME fibre was cleaned and conditioned in a Fibre Bakeout Station in pure nitrogen atmosphere at 250°C for 15 min. The GC/MS analyses were carried out with an Agilent 6890N/5973N GC-MSD (Santa Clara, CA, USA) system equipped with an Agilent SLB-5MS capillary column (30 m × 250 µm × 0.25 µm). The GC oven temperature was programmed to increase from 60°C (3 min isothermal) to 250°C at 8°C min^-1^ (1 min isothermal). High purity helium (6.0) was used as carrier gas at 1.0 mL min^-1^ (37 cm s^-1^) in constant flow mode. The injector was operated in splitless mode at 250°C. The mass selective detector was equipped with a quadrupole mass analyzer and was operated in electron ionization mode at 70 eV in full scan mode (41–500 amu at 3.2 scan s^-1^). The data were evaluated using the MSD ChemStation D.02.00.275 software (Agilent). Identification of compounds was carried out by comparing retention times and recorded spectra with the data of authentic standards, and the NIST 2.0 library was also consulted.

## 3 Results

The antibacterial activity of EOs was evaluated by MIC against the two *Xap* isolates using BMA in 96-well plates. Both *Xap*3 and *Xap*G2 were susceptible to all investigated EOs, but to different degrees (with MIC between 31.25 and 1000 µg/mL), and generally the *Xap*G2 isolate was similar or less sensitive to the EOs than *Xap*3 (**Table 1**). The tea tree EO was the least effective providing the highest MIC value. Comparing the antibacterial potential of EOs to those of antibiotics, the strongest activity was attributed to cinnamon against both *Xap*3 and *Xap*G2 with a 12.5 - and 25 -times higher MIC value than that of chloramphenicol, respectively. In the efficiency order, cinnamon was followed by thyme, clove, and lemon grass with noticeably inhibitory activity.

**Table 1:** Minimal inhibitory concentration (MIC, defined as the lowest concentration at which bacteria suspensions showed no growth) values of EOs and EO constituents tested against two *X. arboricola* pv. *pruni* isolates (*Xap*3 and *Xap*G2).

	MIC (µg/mL)	
Essential oils (EOs)	*Xap*3	*Xap*G2
cinnamon (*Cinnamomum ceylanicum*)	31.25	62.5
thyme (*Thymus vulgaris*)	125	125
clove (*Syzygium aromaticum)*	125	250
lemon grass (*Cymbopogon citratus*)	125	250
lemon balm (*Melissa officinalis* from Olivia Naturals)	250	250
eucalyptus (*Eucalyptus globulus*)	250	250
rosemary (*Rosmarinus officinalis*)	250	500
citronella grass (*Cymbopogon nardus*)	250	500
lemon balm (*Melissa officinalis* from Primavera)	250	500
tea tree (*Melaleuca alternifolia*)	1000	1000
EO constituents		
thymol	50	50
*trans*-cinnamaldehyde	100	100
borneol	200	150
eugenol	150*	400*
citral	400	150
citronellal	400*	400*
terpinen-4-ol	400* <	400* <
		
chloramphenicol	2.5	2.5

^*- the unit is nL/mL^

**Figure 2:**
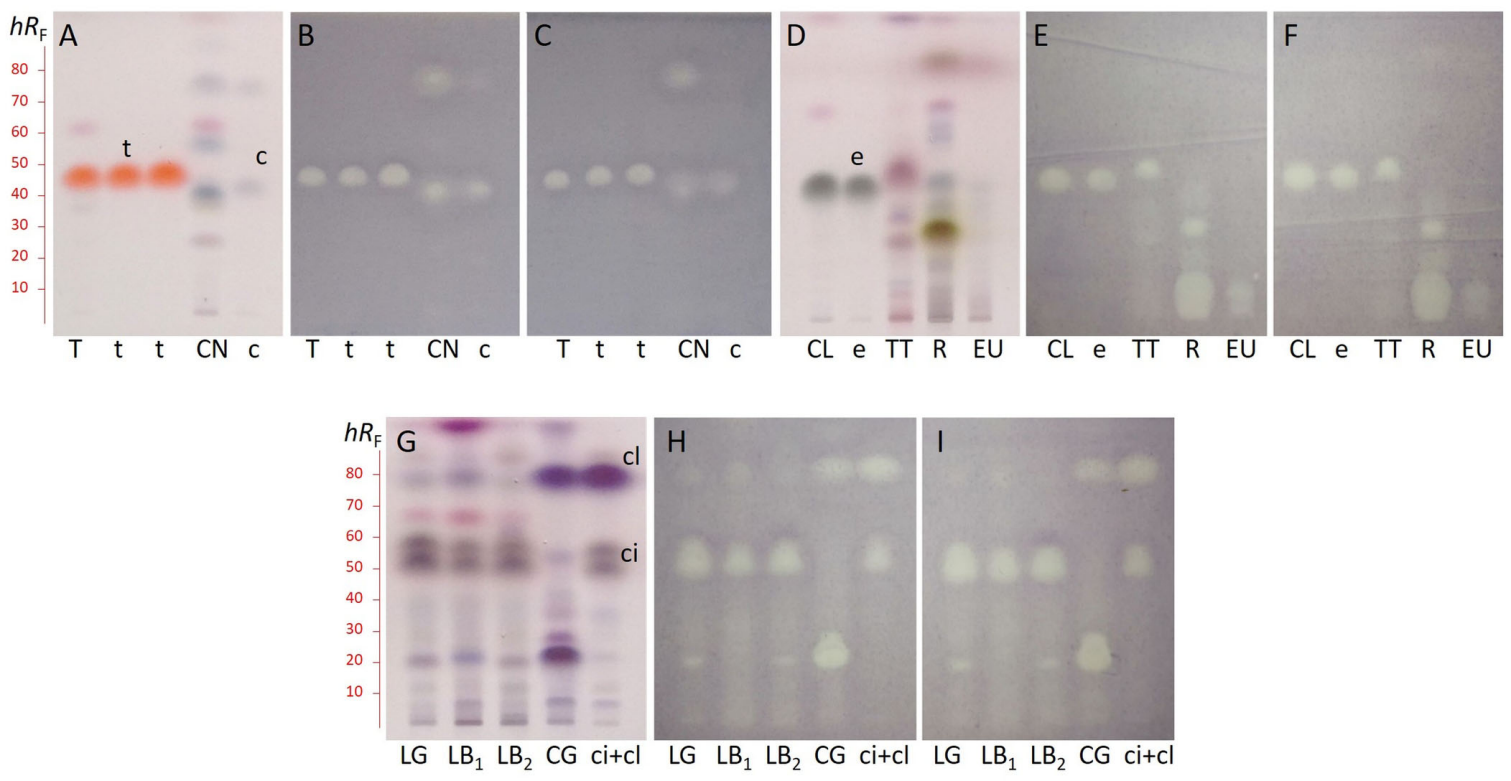
HPTLC chromatograms with retardation factor (*hR*_F_) scale of thyme (T), cinnamon (CN), clove (CL), tea tree (TT), rosemary (R), eucalyptus (EU), lemon grass (LG), lemon balm (LB1 from Primavera, LB2 from Olivia Naturals), and citronella grass (CG) essential oils, and thymol (t), *trans*-cinnamaldehyde (c), eugenol (e), citral (ci: neral+geranial), and citronellal (cl) standards after derivatization with vanillin-sulphuric acid reagent **(A, D, G)**, and bioautograms using *Xanthomonas arboricola* pv. *pruni* isolates *Xap*3 **(B, E, H)** and *Xap*G2 **(C, F, I)**. Bright zones in the bioautograms indicate the bacterial growth inhibition.

To identify the EO components responsible for anti-*Xap* activity, a DB assay was newly adapted for *Xap*. This method enables the detection of separated individual compounds that display *Xap* inhibition. The separation was carried out on HPTLC silica gel 60 F_254_ layer with *n*-hexane – ethyl acetate (9:1, *V*/*V*), and vanillin-sulphuric acid reagent was employed for the visualization of the non-UV active EO compounds (**Figures 2A, D, G**). Generally, the bioprofiles obtained by DB using *Xap*3 (**Figures 2B, E, H**) or *Xap*G2 (**Figures 2C, F, I**) were very similar. In each of thyme and clove EOs, derivatization with vanillin-sulphuric acid reagent revealed one major compound (thymol and eugenol) at a retardation factor (*hR*_F_) 48 and 44 (orange and grey zones) respectively, which gave inhibition against *Xap* isolates (**Figures 2A–F**). Based on the derivatization process, the other EOs were found to be more complex. Among the separated compounds, two in the case of cinnamon at *hR*_F_ 41 and 78 and one in the case of tea tree at *hR*_F_ 51 displayed characteristic antibacterial activities (**Figures 2A–F**). At the zone close to sample application, where separation is questionable, rosemary gave a strong, while eucalyptus a weak inhibition zone (**Figures 2D–F**). In addition, the rosemary component at *hR*_F_ 29 inhibited *Xap* as well (**Figures 2D–F**). In lemon grass and lemon balm the same two compounds at *hR*_F_ 52 and 58 showed antibacterial activities (**Figures 2G–I**). Furthermore, three inhibition zones at *hR*_F_ 23, 29, and 80 appeared in citronella grass (**Figures 2G–I**).

The percentage composition of thyme, cinnamon, clove, and eucalyptus EOs was previously determined in our laboratory by GC demonstrating that the main components are thymol (49.9%), *trans*-cinnamaldehyde (73.2%), eugenol (83.7%), and eucalyptol (84.2%), respectively (Horváth et al., 2013). Thus, not surprisingly, analyzing the compounds in the antibacterial DB zones of these EOs by SPME-GC/MS, thymol (*hR*_F_ 48), *trans*-cinnamaldehyde (*hR*_F_ 41), and eugenol (*hR*_F_ 44) were identified, respectively. In tea tree EO, also the main component terpinen-4-ol (41.3%) was determined in the inhibition zone. The major rosemary components cineole (41.9%) and camphor (18.5%) did not exhibit inhibitory activities, however, borneol (7.7%) was recognized as responsible for the antibacterial effect at *hR*_F_ 29 (**Figures 2D–F**). Neral (*cis*-citral) and geranial (*trans*-citral) were presented in the inhibition zones at *hR*_F_ 52 and 58, respectively (**Figures 2G–I**), which were the constituents of lemon grass and lemon balm EOs with the highest percentage (each between 32% and 39%). Compounds in antibacterial zones of citronella grass EO at *hR*_F_ 23 and 80 were identified as nerol and citronellal that have the highest abundance in the oil with 17.3% and 37.6%, respectively (**Figures 2G–I**).

The antibacterial activity of the compounds identified by the combination of HPTLC-*Xap* assays and SPME-GC/MS was assessed also by MIC (**Table 1**). The anti-*Xap* effect of all compounds was confirmed (with MIC between 50 and 400 µg/mL or nL/mL), except for terpinen-4-ol that did not inhibit the bacterial growth in the applied concentrations. The two *Xap* isolates showed similar sensitivity to EO components, but citral and borneol were more effective on *Xap*G2 than on *Xap*3, while the *Xap*3 isolate was more sensitive to eugenol compared to *Xap*G2. The highest inhibitory activity was attributed to thymol with a MIC of 50 µg/mL.

## 4 Discussion

Importantly, results of relevant studies on antibacterial effects of EOs are difficult to compare, not only because of differences in applied methods, but also because of the variable composition of EOs, influenced by geographical, climatic, edaphic and seasonal factors (Ložienė and Venskutonis, 2005; Aboukhalid et al., 2017; Tsiftsoglou et al., 2023). Regarding *Xap*, our results enable comparing antimicrobial activities of lemon grass and eucalyptus EOs to literature data. The *Xap* isolates tested in this study (*Xap*3, *Xap*G2) showed higher sensitivity (with MIC values of 125 and 250 µg/mL) to lemon grass EO than the *Xap* strain examined by Zhao et al. (2011) (MIC 500 µL/L). Montesinos et al. (2023) reported a reduced susceptibility of *Xap* (MIC 10 – 30 µl/mL) and other species such as *X. fragariae* and *X. axonopodis* pv. *vesicatoria* (MIC < 5 and 10 – 30 µl/mL, respectively) to eucalyptus EO, as compared to our observations, however they used a different method, the agar incorporation test. The effectiveness of other EOs on *Xap* has been previously also revealed; antibacterial effects of *Thymbra sintenisii* and *Satureja cuneifolia* EOs were reported with MIC values of 600 ppm and 800 ppm, respectively (Biavati et al., 2004). Furthermore, Mahmoudi et al. (2010) investigated the antibacterial activities of fourteen EOs and four plant extracts on *Xap* using the agar diffusion method and they have found peppermint, cumin, slender ziziphora, bishop’s weed, common sage and neem tree to be the most effective.

Effects of EOs on other *Xanthomonads* are widely known; Mačionienė et al. (2021) have found that *T. vulgaris* EO had the strongest antibacterial effect against *X. translucens* and five tested *X. arboricola* strains (unindicated pathovars) with 7.81 µg/mL MIC value as revealed by BMA. However, in case of the Hungarian *Xap* strains, the *T. vulgaris* EO provided a higher MIC value (125 µg/mL). Kotan et al. (2007) investigated the effect of EOs of other *Thymus* spp. on the growth of *X. axonopodis* pv. *vesicatoria* by the agar diffusion method, and found that the test bacterium was the most sensitive to *T. sipyleus rosulans* (MIC 25 µg/mL) out of the other *Thymu*s spp. with 200 µg/mL MIC value. Testing the antibacterial efficacy of another thyme species by BMA, the *T. spathulifolius* EO was effective against *X. campestris*-A235 strain at a higher concentration (MIC 500 µg/mL) (Sokmen et al., 2004). Gormez et al. (2016) reported remarkable results testing *Origanum rotundifolium* EO - whose main compounds are carvacrol and thymol - against *X. vesicatoria* and *X. axonopodis* pv. *campestris* with 7.81 and 31.25 µg/mL MIC values, respectively, using BMA. Mirzaei-Najafgholi et al. (2017), applying BMA as well, found that *Citrus aurantifolia* EO exhibited a higher activity on *X. citri* ssp. *citri*, as compared to that of *Citrus aurantium* EO with 500 and 1300 µg/mL MIC values, respectively. As for the active ingredients of EOs, Kotan et al. (2007) also investigated the effect of thymol. However, the *X. axonopodis* pv. *vesicatoria* strain gave a much lower MIC value (3.125 µg/mL) against thymol than the Hungarian *Xap* strains in our experiments (50 µg/mL) suggesting that the given *X. axonopodis* pv. *vesicatoria* strain is more sensitive to the main active compound of thyme. *Cleistocalyx operculatus* EO also displayed significant antibacterial activity against four *Xanthomonads* with MIC values of 31.25–125 μg/mL in BMA (Bajpai et al., 2010). Importantly, although the effects of EOs including that of thyme against *X. campestris* pv. *vesicatoria* and *X. euvesicatoria* was investigated by direct bioautography (Horváth et al., 2004; Móricz et al., 2015a; Móricz et al., 2015b), to our knowledge, this method has not yet been introduced against *Xap*.

The bioactivity and action mechanism of EOs strongly depends on their composition. EOs are constituted of volatile secondary metabolites including mono-, and sesquiterpenes, as well as oxygenated compounds, like alcohols, phenols, aldehydes, and esters (Hyldgaard et al., 2012; Raveau et al., 2020; Angane et al., 2022). Terpenes of EOs, such as cinnamon or clove EO, are able to disrupt and penetrate the lipid structure of the cell wall and the bacterial cell membrane, leading to protein denaturation and cell membrane destruction, cytoplasmic leakage, pH decrease, loss of DNA transcription, protein synthesis and enzyme activity, cell lysis, and ultimately cell death (Oussalah et al., 2006; Raybaudi-Massilia et al., 2006). In addition, cinnamon EO and its components have been showed to inhibit amino acid decarboxylases in Gram-negative bacteria (Wendakoon and Sakaguchi, 1995). Similar to other phenolic compounds, thymol or eugenol is assumed to damage the structure and function of the cytoplasmic membrane, which disrupts the proton motive force, the electron flow, and active transport causing coagulation of cell contents (Sikkema et al., 1995). Terpenoids of lemon grass also permeabilize the cell membrane (Hyldgaard et al., 2012; Nguefack et al., 2012).

In fact, the antimicrobial activity of EOs is assigned to a number of small terpenoids and phenol compounds (*e.g*. thymol, carvacrol, eugenol, cinnamaldehyde), which display high antibacterial activities also in pure form (Conner, 1993; Didry et al., 1993; Horváth et al., 2004). However, the efficacy of EOs may not exclusively depend on the ratio of the main active compounds, but also on interactions between active compounds and their minor constituents (Hyldgaard et al., 2012). In such multicomponent mixtures, the possible occurrence of interactions among the components can result in synergistic, additive, or antagonistic effects (Hakalová et al., 2022; Połeć et al., 2022). Thus, similar to our results obtained with cinnamon, clove, lemon grass, lemon balm, and citronella grass EOs, some studies have demonstrated that whole EOs have greater antibacterial activity than the major components (Mourey and Canillac, 2002; Burt, 2004). This synergism may also underlie the fact that cinnamon EO, which exhibited the highest efficacy in our experiments, had a lower MIC than that of its main active ingredient *trans*-cinnamaldehyde.

To our knowledge, no EO-based antibacterial product have been registered in the EU so far, however, three other types of biopesticides are available; the Mevalone^®^ biofungicide containing geraniol, eugenol, thymol against *Botrytis cinerea* in wine grapes, the Bioxeda^®^ post-harvest fungicide with clove EO as an active ingredient against *Gloeosporium* spp. and *Penicillium* spp. on apples, peaches and pears and PREV-AM^®^ containing orange EO to control whiteflies on tomatoes and zucchini (EC, 2023). In addition, it is important to mention that an EO-based biopesticide against *Xap* is already available in the USA: Guarda^®^ with the active ingredient thyme EO formulated by HOLDit^®^ technology is recommended against *Xap* besides many fungal and bacterial pathogens (BioSafe Systems, 2023). Applications of EOs as alternative biopesticides under field conditions are confronted by the loss of efficacy or performance due to their volatility, instability, low solubility in water, different viscosity, and aptitude for oxidation (Pavoni et al., 2020). Therefore, EOs require appropriate formulation to preserve their bioactivity and to extend and control their release time. Encapsulation of pesticides is usually ensured by solid nanoparticulate or liquid nanoemulsion systems with the aid of natural polymers (*e.g.* polysaccharides), wettable powders or granules, surfactants, stabilizers, liposomes and solvents (Benita, 2006; Borges et al., 2018; Pavoni et al., 2020). Such nanocarrier systems stabilize the EOs, along with improving bioavailability and amplifying antimicrobial efficacy (Anwer et al., 2014; Micucci et al., 2020; Wen et al., 2021). Nanoencapsulation of thyme and cinnamon EOs and their main constituents (thymol and cinnamaldehyde, respectively) in nanocarriers were found to be a promising approach for development of biopesticides (Falleh et al., 2021; Sousa et al., 2021; Abdelhamed et al., 2022; Alsakhawy et al., 2022). In comparison to pure EO, nanosponges of cinnamon EO prepared with ethyl cellulose and polyvinyl alcohol had significantly higher antibacterial effects against *Staphylococcus aureus* (Kaur et al., 2020). Similarly, a nanostructured lipid carrier cinnamon gel had about 10 times higher antibacterial efficacy against *P. aeruginosa* than the EO alone (Wen et al., 2021), and the effective concentration of cinnamaldehyde to control plant disease caused by *Pseudomonas syringae* pv. *pisi* became 90,000-fold lower by a formulation with silica nanoparticles (Bravo Cadena et al., 2018). Furthermore, the nanoemulsion with *Thymus daenensis* EO, an abundant source of thymol and carvacrol, revealed enhanced antibacterial activity against *E. coli* compared to the pure EO (Moghimi et al., 2016). In summary, the advanced technology formulation that enables increased stability, efficiency and water solubility could be the key for the widespread application of EOs as antibacterial plant protection agents (Das et al., 2020; López et al., 2021; Shankar et al., 2022; Singh and Pulikkal, 2022).

To our knowledge, this is the first report on the antibacterial activity of these EOs (except for lemon grass and eucalyptus) and their major constituents against *Xap*. Moreover, the HPTLC-*Xap* (DB) method was introduced to identify anti-*Xap* EOs’ ingredients. Our results suggest that out of the ten EOs tested, the most effective ones - cinnamon, thyme, clove and lemon grass - have the potential to be promising candidates for alternative biopesticides to control the plant disease caused by *Xap*.

## Data availability statement

The original contributions presented in the study are included in the article/supplementary materials. Further inquiries can be directed to the corresponding authors.

## Author contributions

ÁMM and IS: conceptualization, methodology and supervision; JKN, ÁMM, AB, ÁA, and IS: Investigation and data analysis; JKN, ÁMM, and IS: writing - review and editing; ÁMM: visualization, funding, project administration. All authors contributed to the article and approved the submitted version.
